# Long term survival achieved through combination of almonertinib and pyrotinib in EGFR-mutant/HER2-amplified advanced NSCLC patient: a case report and literature review

**DOI:** 10.3389/fonc.2024.1397238

**Published:** 2024-08-09

**Authors:** Xin Pan, Xiao Zhou

**Affiliations:** Department of Oncology, Tongji Hospital, Huazhong University of Science and Technology, Wuhan, Hubei, China

**Keywords:** HER2 amplification, EGFR mutation, almonertinib, pyrotinib, NSCLC

## Abstract

**Backgroud:**

Human epithelial growth factor receptor 2 (HER2) amplification is an important mechanism of acquired resistance to anti-epidermal growth factor receptor (EGFR) therapy in non-small cell lung cancer (NSCLC) patients. For patients with both EGFR mutation and HER2 amplification, there is currently no unified standard treatment, and further exploration is needed on how to choose the therapy.

**Methods and results:**

A female NSCLC patient developed bone and brain metastases 14 and 42 months after radical surgery, respectively. The second genetic sequencing detected EGFR L858R mutation and HER2 amplification, and therefore initiated treatment with almonertinib and pyrotinib. The patient achieved partial remission and did not show any further progression during the follow-up period.

**Conclusion:**

For NSCLC patients with both EGFR mutation and HER2 amplification, the combination of almonertinib and pyrotinib is a valuable therapy that can continuously reduce tumor burden and achieve long-term survival.

## Introduction

In recent years, the development of molecular targeted therapy has improved the clinical outcomes of NSCLC patients (1, 2). About 50% of Asian nonsmoking NSCLC patients have EGFR activating mutations, with exon 19 deletion and L858R mutation accounting for 80-90% of cases. L858R mutation is the substitution of leucine to arginine in exon 21 of EGFR, representing 40% of all mutations (3). EGFR-tyrosine kinase inhibitors (TKIs) are commonly used as the first-line treatment for advanced NSCLC patients with EGFR mutations, and has been shown to prolong survival (4). Although initially showing benefits, EGFR-mutant NSCLC patients will eventually develop resistance to treatment with EGFR-TKIs (5). The 5-year overall survival (OS) rate of NSCLC patients is still below 20% (2).

The resistance mechanisms of EGFR-TKIs can be broadly divided into primary and acquired resistance. There are two mechanisms for acquired resistance to EGFR-TKIs.

EGFR-dependent mechanism involves secondary mutations in EGFR. EGFR-independent mechanism includes activation of alternative bypass or downstream pathways and histological transformation (6). For the first- and second- generation EGFR TKIs, the secondary T790M mutation is the most commonly mechanism of EGFR-dependent resistance, accounting for approximately 50-60% of all cases. However, for patients treated with the third-generation EGFR TKIs, only 10-20% exhibit EGFR-dependent resistance, and the C797S mutation symbolizes the main mechanism of EGFR-dependent resistance (7). EGFR-independent acquired resistance caused by activation of alternative bypass or downstream pathways mainly contains HER2 amplification, MET amplification, KRAS mutations, BRAF mutations, PIK3A mutations and so on (8–12). HER2 has no known corresponding ligand and activates downstream signaling pathways by forming homodimers or heterodimers with other EGFR family proteins (13). The amplification and oncogenic mutations of HER2 in lung cancer promote hyperactivation of downstream PI3K–AKT and MEK–ERK/MAPK pathways, promoting carcinogenesis (13, 14). Recent studies have reported the important role of HER2 genetic alterations in acquired resistance to EGFR-TKIs (8, 15). HER2 amplification is one of the driving factors and potential therapeutic targets for lung cancer. However, there is currently no consensus on the optimal treatment for HER2-amplified NSCLC patients. Pyrotinib is a pan ErbB receptor tyrosine kinase inhibitor targeting HER1/2/4, which inhibits HER2 phosphorylation by blocking the homodimers or heterodimers binding of the EGFR family to HER2, thereby preventing the activation of downstream signaling pathways, and thus inhibiting tumor cell growth (16). PHILA is a randomized, double-blind, parallel controlled, multi-center phase III clinical trial. The research results indicate that the pyrotinib combined with trastuzumab and docetaxel has promising efficacy as first-line treatment for HER2-positive recurrent or metastatic breast cancer patients. The median progression-free survival (mPFS) of the pyHT treatment group lasted for 24.3 months (HR=0.41, 95% CI 0.32-0.53; P<0.0001) (17). Pyrotinib combined with trastuzumab and docetaxel significantly reducing the risk of disease progression and death of patients, and creating a new pattern of first-line treatment of HER2-positive advanced breast cancer. In the second-line treatment for HER2-positive advanced breast cancer, pyrotinib showed satisfactory effect in prolonging PFS. The PHENIX trial reported that the mPFS of patients with HER2-positive advanced breast cancer who failed to trastuzumab treatment in the pyrotinib plus capecitabine group and the placebo plus capecitabine group is 11.1 months and 4.1 months (HR=0.18, 95%CI 0.13-0.26; P<0.001), respectively (18). Therefore, pyrotinib combined with capecitabine has been approved as the second-line treatment for HER2-positive advanced breast cancer. Currently, a few completed and ongoing clinical research indicate that pyrotinib is a promising drug for HER2-altered NSCLC patients. For stage IIIB or IV HER2-mutant lung adenocarcinoma patients who have previously received platinum chemotherapy, the objective effective rate (ORR) of pyrotinib therapy is 30.0% (95%CI 18.8%-43.2%), the mPFS is 6.9 months (95%CI 5.5-8.3), and the mOS is 14.4 months (95%CI 12.3- 21.3) (19). ChiCTR1800020262 trial suggested that the 6-month incidence of PFS in HER2-amplified advanced NSCLC patients treated with pyrotinib is 51.9% (95%CI 34.0%- 69.3%). The other endpoints of this study included the mPFS of 6.3 months (95% CI 3.0-9.6), the mOS of 12.5 months (95% CI 8.2-16.8), and the ORR of 22.2% (95% CI 10.6-40.8%) (20).

This article presents the treatment process of an EGFR-mutant/HER2-amplified NSCLC patient, and summarizes the latest progress in HER2 targeted therapy for this population. To our knowledge, this is the first description of the combined use of almonertinib and pyrotinib in EGFR-mutant/HER2-amplified NSCLC patients. HER2 amplification represents up to 10% of acquired resistance to EGFR-TKIs, while HER2 amplification only accounts for 3% in NSCLC patients without EGFR-TKI therapy (8). Our report, together with the clinical outcome of a patient from other centers, suggests that the combination of the third-generation EGFR-TKIs and pyrotinib has the potential to achieve long-term survival in EGFR-mutant/HER2-amplified NSCLC patients.

## Case presentation

The patient was a 50-year-old woman. She was admitted to her local hospital for a routine physical examination on 28 May 2019. The computerized tomography (CT) scan showed a soft tissue mass of approximately 2.5 cm × 3 cm in the upper lobe of the left lung. There were no significant abnormalities found in the abdomen, the head or the bones. When inquiring about her condition, she denied any symptoms such as hoarseness, cough, hemoptysis, chest pain or dyspnea. She had no previous medical history and no notable personal and family history. Physical examination revealed normal breath sounds in both lung fields. Laboratory tests showed that the complete blood count and blood biochemical indicators were within the normal range.

The patient agreed to undergo surgery after digesting the information we provided. A thoracoscopic left upper lobe lobectomy was performed in the local hospital on 01 June 2019. Postoperative pathological examination indicated: left lung adenocarcinoma (solid type 90%, micropapillary 5%), no involvement of pleura, bronchial stump and anastomotic nail stump, no neurovascular invasion, no parbronchial lymph node invasion (0/3), station 6 lymph nodes (1/7), station 7 lymph nodes (0/12), station 10 lymph nodes (0/1), station 11 lymph nodes (0/4). The tumor was classified as pT1cN2M0, Stage IIIA according to the TNM classification of the Union of International Cancer Control (UICC), 6th edition. Genetic testing suggested EGFR (-), ALK (-), and ROS1 (-). Then she underwent a chemotherapy consisting of pemetrexed and cisplatin for four cycles from 25 August 2019 to 08 November 2019, followed by intensity-modulated radiation therapy (IMRT) with 50 Gy to the clinical tumor volume (CTV).

**Figure 1:**
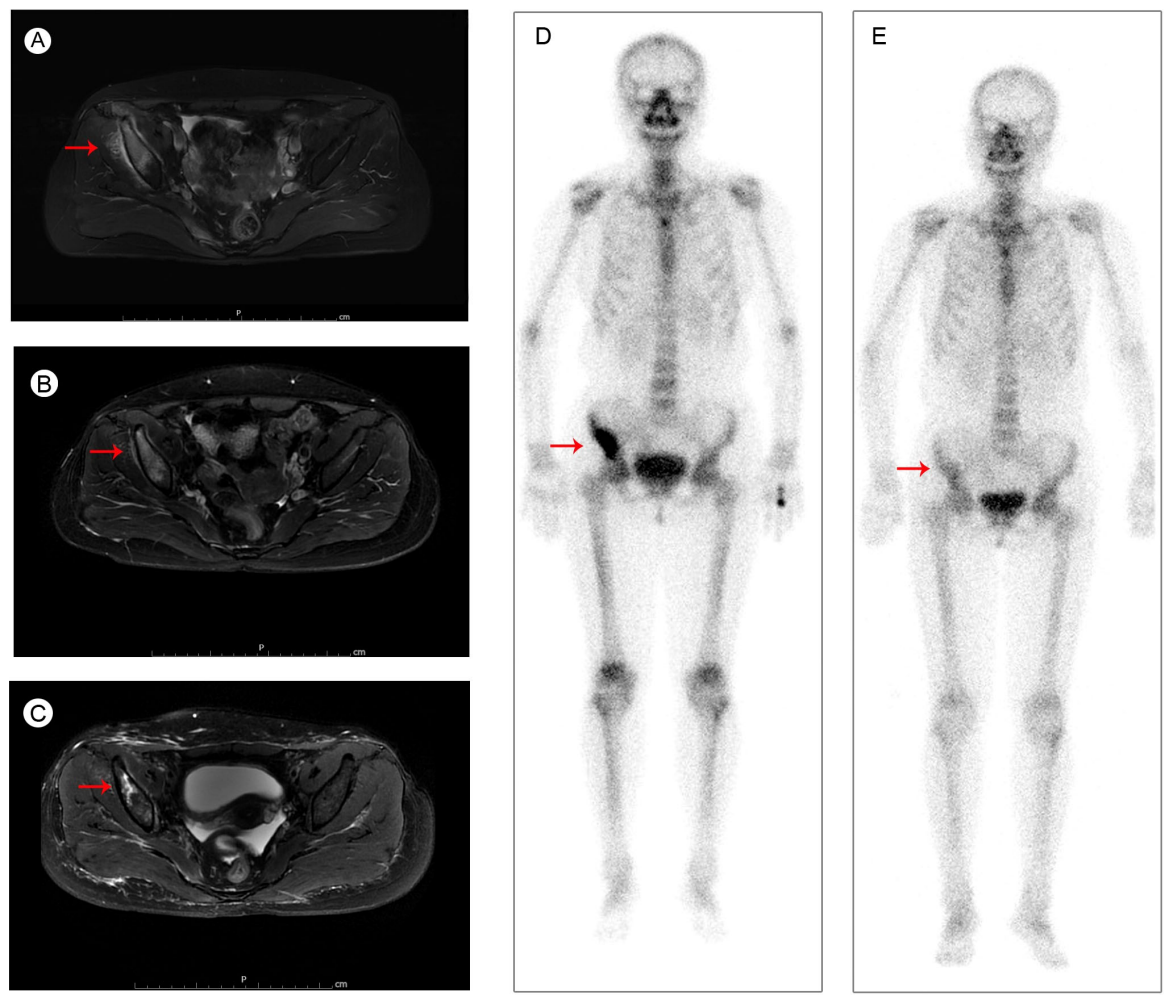
Pelvic MRI images of iliac bone metastases **(A)** before radiotherapy in August 2020; **(B)** after radiotherapy in September 2020; **(C)** during the albumin-bound paclitaxel treatment in September 2021; and bone scan images **(D)** before radiotherapy in August 2020; **(E)** during the albumin-bound paclitaxel treatment in September 2021.

The patient felt pain and discomfort in the right buttock half a year later and came to our hospital for further diagnosis and treatment on 05 August 2020. Her magnetic resonance (MR) scanning images and bone scan image showed abnormal signals in the right sacrum, ilium and acetabulum, considering tumor metastasis (**Figures 1A, D**). CT scan of the chest and abdomen showed no significant abnormalities in other parts. Then she received palliative radiotherapy (30 Gy in 10 fractions) for bone metastases. The pain in her buttock was significantly relieved after radiotherapy. And the tumor size was obviously reduced (**Figure 1B**). Beginning in 29 August 2020, the patient received albumin-bound paclitaxel for one year. According to the Response Evaluation Criteria in Solid Tumors (RECIST) version 1.1, she experienced stable disease (SD) during this period (**Figures 1C, E**). Due to intolerable adverse effects (numbness and pain in the hands and feet) and insufficient treatment costs, the patient no longer agreed to received albumin-bound paclitaxel. Her treatment regimen was changed to anlotinib (a multi-targeting TKI) in December 2021.

**Figure 2:**
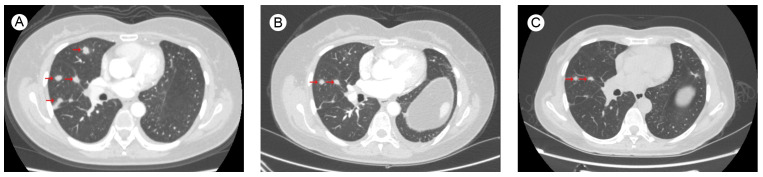
Chest CT images of lung metastases **(A)** before Almonertinib + Pyrotinib treatment in December 2022; during Almonertinib + Pyrotinib treatment **(B)** in March 2023; and **(C)** in November 2023.

**Figure 3:**
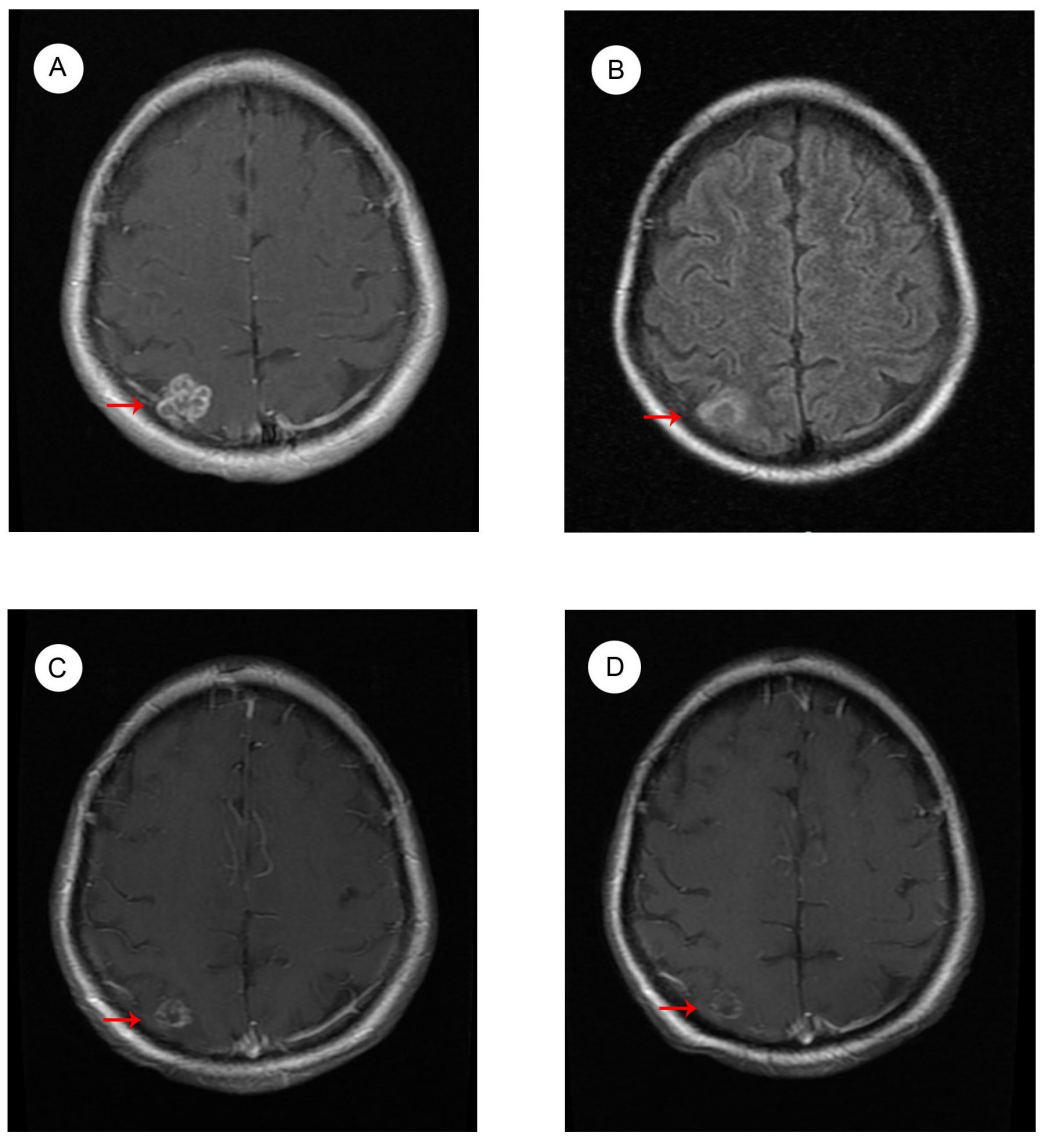
MRI images of brain metastases **(A)** before radiotherapy in December 2022; **(B)** after radiotherapy in January 2023; during Almonertinib + Pyrotinib treatment **(C)** in March 2023; and **(D)** in November 2023.

**Figure 4:**
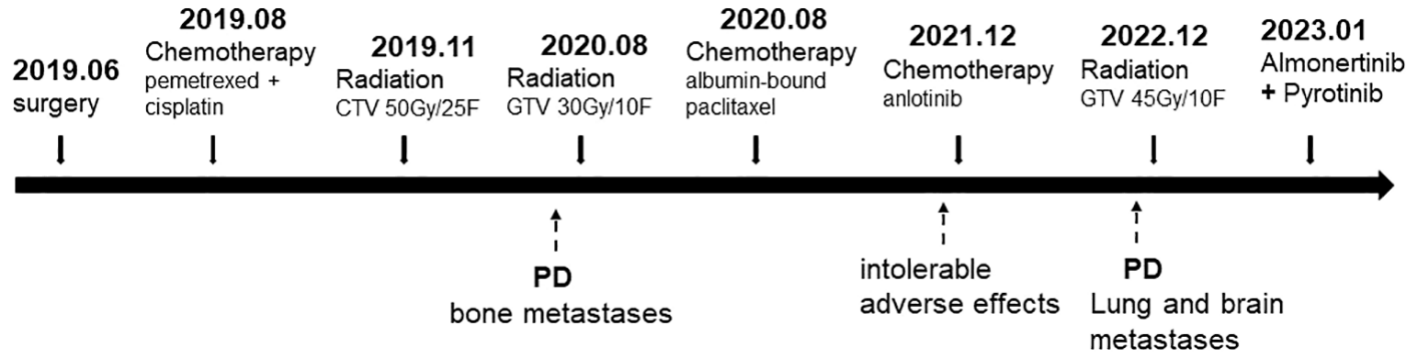
Summary of the treatment timeline of this case.

The patient started to experience dizziness in December 2022. A chest CT scan revealed multiple nodules in both lungs, increased and partially enlarged lymph nodes in the right hilar and mediastinal lobes, which were considered metastatic tumors (**Figure 2A**). MRI of the brain revealed strengthened nodules in the right temporal lobe and parietal cortex (**Figure 3A**). Then the patient underwent IMRT for the brain tumor (45 Gy in 10 fractions) on 20 December 2022. The symptoms of headache and dizziness were significantly relieved after radiotherapy. MRI showed the tumor size and number were obviously reduced (**Figure 3B**). We recommended that she undergo whole-genome sequencing. The patient refused re-puncture for sampling. Only peripheral blood was used for genetic testing and detected EGFR L858R mutation and HER2 amplification. Therefore, chemotherapy with almonertinib (HS-10296; a third-generation EGFR-TKI) and pyrotinib (a pan-ErbB receptor TKI) was administered in January 2023. Two months later, the CT scan of the chest showed that the nodules in both lungs were significantly reduced (**Figure 2B**). The MRI revealed stabilization of the disease in the brain (**Figure 3C**). The main adverse event was grade II diarrhea (according to the common terminology criteria for adverse events of the National Cancer Institute of the United States, CTCAE 5.0). As of the last review in November 2023, the patient’s efficacy was evaluated as partial response according to the RECIST version 1.1 (**Figures 2C**, **3D**). The timeline for treatments and efficacy evaluations in this case is summarized in **Figure 4**.

## Literature review

### Prevalence of HER2 amplification in NSCLC

HER2 is a transmembrane receptor tyrosine kinase (RTK). The three main forms of HER2 activation are gene mutations, gene amplification, and protein overexpression (21). Current studies typically use next generation sequencing (NGS) to detect HER2 mutations. HER2 amplification and expression was assessed by FISH and IHC. According to the HER2 detection guidelines in breast cancer issued by the American Society of Clinical Oncology/College of American Pathologists, HER2 amplification is defined as HER2/chromosome enumeration probe 17 [CEP17] ratio ≥ 2.0 (22). The current reported proportion of HER2 amplification in NSCLC is 3%-19% (23–25). A retrospective analysis showed that HER2 amplification was observed in 14.3% of lung adenocarcinoma patients, with a higher proportion of male smokers and pleural invasion. Besides, HER2 amplification is a poor prognostic factor for lung adenocarcinoma, associated with shorter overall survival and disease-free survival. HER2 amplification leads to a higher incidence of brain metastasis and pleural invasion in NSCLC (23). Data published by the Memorial Sloan Kettering Cancer Center and the University of Colorado in 2016 showed that HER2 amplification was detected in 5 out of 175 lung adenocarcinoma patients (3%) without targeted therapy, while HER2 mutations were detected in 4 out of 148 patients (3%). Interestingly, this study found that HER2 amplification and mutation are mutually exclusive (26). HER2 amplification usually does not occur simultaneously with the mutation in lung adenocarcinoma patients (14, 27). Although recent studies have reported cases with both HER2 amplification and mutations, this overlap is rare (24, 25, 28). Suzuki et al. reported that out of 1170 NSCLC patients, 222 cases (19.0%) detected HER2 amplification, which is much higher than other studies (25). It should be noted that in the above studies, the assessment of HER-2 amplification was performed by different methods and using different criteria, which may have implications on study findings and interpretation.

### Effect of HER2 amplification on EGFR‐targeted therapy in NSCLC

EGFR mutation is a representative mutated pattern in NSCLC. At least 80% of EGFR mutations occurring in NSCLC are characterized by the deletion of the conserved sequence E746-A750 in exon 19 and the substitution of leucine to arginine at position 858 (3). EGFR-TKIs as first- and second-line treatment have improved the prognosis and quality of life of advanced lung cancer patients, but inevitably lead to drug resistance. The acquired resistance mechanism of EGFR-TKIs can be divided into EGFR-dependent and EGFR-independent mechanism. EGFR-independent mechanisms include activation of alternative pathway and histological transformation. HER2 amplification is considered one of the oncogenic driving factors of lung cancer and an important mechanism of EGFR-independent mechanisms of acquired resistance to EGFR-TKIs. HER2 amplification accounts for 2% and 5% of drug-resistant mutations after first-line and second-line treatment with Osimertinib, respectively (15, 29). Among the drug-resistant mutations after first- and second-generation EGFR-TKIs, the proportion of HER2 amplification is relatively high, at 10% (8). HER2 amplification typically occurs in subpopulations of NSCLC lacking EGFR T790M mutation (8). In terms of tumor immune microenvironment, NSCLC patients with HER2 amplification have a higher tumor mutational burden (TMB) than those with HER2 mutation, while no difference in PD-L1 expression was observed between the two subgroups. Compared with primary HER2 amplification, acquired HER2 amplification that occurs after EGFR-TKIs resistance has lower TMB and PD-L1 expression (30). *In vitro* studies have shown that HER2 amplification reduces the anti-tumor activity of Osimertinib on lung cancer cells (31). These *in vivo* and *in vitro* studies reveal the role of HER2 amplification in EGFR-TKIs resistance and provide a theoretical basis for evaluating the possibility of targeting HER2 therapy in EGFR-mutant NSCLC patients with acquired resistance to EGFR-TKIs.

### Treatment trend for NSCLC patients with HER2 amplification

Chemotherapy is the preferred treatment for HER2-amplified NSCLC, with a mPFS for 5-6 months (32). Targeted HER2 therapies have shown limited clinical efficacy in HER2-amplified NSCLC patients. Therefore, there is no approved targeted therapy for HER2-amplified NSCLC patients. The Phase II study of trastuzumab targeting HER2 protein expression positive patients did not show significant benefits (33). Despite the early setbacks, clinical trials of anti-HER2 treatment in lung cancers are currently underway. For NSCLC patients, targeted HER2 therapies currently in clinical trials include monoclonal antibodies (trastuzumab and pertuzumab), TKIs (afatinib, pyrotinib, dacomitinib and neratinib), and antibody-drug conjugates (ADCs) (T-DM1, T-DXd). There are few reports on the efficacy and safety of pyrotinib in NSCLC patients with HER2 alteration in the real world. ChiCTR1800020262 is a prospective, multicenter, single-arm trial investigating the efficacy of pyrotinib in 27 NSCLC patients with HER2-amplified. In the study, patients who failed treatment with EGFR-TKI responded to pyrotinib with an objective response rate (ORR) of 30.8% and mPFS of 7.2 months. Pyrotinib can penetrate the blood-brain barrier, and HER2-amplified NSCLC patients with brain metastases can benefit from it. HER2-amplified NSCLC patients have the same clinical outcomes as those with HER2-amplified/mutant, indicating that concurrent HER2 mutation did not affect the anti-tumor activity of pyrotinib (20). This is the first prospective clinical study to demonstrate the curative effect and controllable safety of pyrotinib in NSCLC patients with HER2-amplified, limited by the absence of randomized controls and the absence of FISH validation of HER2-amplified status. Another retrospective study showed that pyrotinib was safe and well tolerated in NSCLC patients with HER2 alterations. The most common level three or higher adverse events are diarrhea and leukopenia, which can be controlled through drug reduction and adjuvant therapy to maintain treatment (34). A case report in 2021 showed that patients with both EGFR mutations and HER2 amplification can benefit from combination therapy targeting EGFR and HER2. This is the first description of the clinical efficacy of osimertinib combined with pyrotinib in the treatment of EGFR mutant/HER2 amplificated NSCLC (35). The anti-tumor effect of anti-HER2 ADCs on NSCLC patients is not related to the expression level of HER2 protein. Two clinical trials exploring the efficacy of ADCs in HER2-overexpressed lung cancer patients did not achieve satisfactory clinical outcomes. But in populations with HER2-amplified/mutant, anti-HER2 ADCs produced the expected effect. The result of the NCT02675829 Phase II clinical study showed that the objective response rate of T-DM1 for 49 lung cancer patients with HER2 amplification and/or mutation was 51%, and the mPFS was 5 months. Whether HER2 amplification or mutation occurs in these lung cancer patients, there is no appreciable differences in the efficacy of T-DM1. Of the 49 lung cancer patients included in this study, six had both HER2 amplification and EGFR mutations. All of them developed disease progression after treatment with EGFR inhibitors, and two of them responded to T-DM1 (36). At present, T-DXd is recommended as the second-line treatment for HER2 mutant NSCLC. However, the occurrence of related adverse events such as interstitial pneumonia and the high cost limit its clinical application. The promotion of current clinical research results is limited by insufficient cases, nonrandomized controls, and inconsistent evaluation methods for HER2 amplification. However, these results provide a promising combination therapy for NSCLC patients who have failed EGFR-TKI treatment with HER2 amplification. It is necessary to conduct large-scale prospective randomized clinical trials in the future.

## Discussion

At present, chemotherapy is the preferred option for advanced NSCLC patients with HER2 alterations who are not suitable for targeted therapy, and its mPFS as first-line therapy is 6 months. The optimal choice for HER2 targeted therapy in the treatment of NSCLC is still unclear. Targeted therapy against HER2 alone is insufficient to generate strong and lasting anti-tumor response in NSCLC patients with EGFR mutant and HER2 amplification. Therefore, further research is needed to explore effective targeted therapy for this group of patients. Our case suggests that advanced NSCLC patients with both EGFR mutation and HER2 amplification may achieve sustained disease control through the combination of almonertinib and pyrotinib, possibly due to the simultaneous inhibition of the EGFR and HER2 signaling pathways. At the same time, our report also has some limitations and shortcomings. Our report requires further extension of follow-up time to better demonstrate the efficacy of the combination therapy. In general, our case report may be useful for clinical protocol selection. It is necessary to conduct prospective trials focusing on combination of EGFR TKIs with pyrotinib to provide more valuable evidence for reference.

## Data Availability

The original contributions presented in the study are included in the article/supplementary material. Further inquiries can be directed to the corresponding author.
